# Cold-Adapted Uric Acid-Degrading *Lacticaseibacillus paracasei* NEFU-6 Application in Kimchi “*Paocai*”

**DOI:** 10.3390/molecules31101717

**Published:** 2026-05-18

**Authors:** Xiaoyu Wang, Binyu Cui, Xiaoqian Zhou, Wei Zhang, Aman Khan, Weidong Wang

**Affiliations:** 1 Key Laboratory of Green and Low-Carbon Agriculture for Northeastern Plains, Ministry of Agriculture and Rural Affairs, College of Life Science and Technology, Heilongjiang Bayi Agricultural University, Daqing 163319, China; wangxiaoyu12025@126.com; 2 College of Life Sciences, Northeast Forestry University, Harbin 150400, Chinaaman@lzu.edu.cn (A.K.)

**Keywords:** Kimchi, probiotics, uric acid degradation, cold-adapted, *Lacticaseibacillus paracasei*

## Abstract

The use of lactic acid bacteria for the management of hyperuricemia has attracted growing interest, whereas the specific emphasis on cold-adapted uric acid-degrading probiotics in the fermentation of traditional foods remains underexplored. In this study, *Lacticaseibacillus paracasei* NEFU-6 was isolated from Northeastern Chinese Kimchi and efficiently degraded uric acid (UA) at a temperature relevant to food fermentation (15 °C) and under simulated physiological conditions (37 °C). The results showed that strain NEFU-6 degraded 25.48% of UA in 6 days at 15 °C, and 40.55% after 72 h at 37 °C in 0.84 g/L of uric acid. All probiotic and safety-related properties were evaluated at 37 °C to simulate human physiological conditions. In vitro probiotic characterization revealed that strain NEFU-6 exhibits non-hemolytic activity, strong free radical-scavenging capacity, significant surface hydrophobicity, and an auto-aggregation rate of 52.65% after 24 h. The strain NEFU-6 also demonstrated robust survival under simulated gastrointestinal conditions, with tolerance rates of 70.7% in 0.3% bile salts, 51.02% in gastric juice at pH 1.5, and 62.61% after 4 h of exposure to artificial intestinal fluid, indicating strong adaptability. Furthermore, the application of strain NEFU-6 in kimchi fermentation improved product quality, confirming its potential for the development of low-temperature functional foods.

## 1. Introduction

Uric acid (UA), the oxidative metabolite of purine compounds in humans, can accumulate to excessive levels and lead to hyperuricemia [1]. Elevated serum UA levels characterize this condition and is a major risk factor for gout and various other metabolic disorders [2]. UA is inherently linked to a complex network of precursor metabolites, including hypoxanthine, xanthine, inosine, and various nucleotides, making its regulation highly dependent on the broader purine metabolic pathway [3]. Emerging evidence indicates significant dysbiosis in the gut microbiota of individuals with hyperuricemia compared with healthy individuals, suggesting that the intestinal microbiome is a key modulator of systemic UA levels [4].

Within this context, lactic acid bacteria (LAB), a prominent group of probiotics, have attracted growing research interest for their ability to regulate UA through two principal mechanisms. First, LAB can secrete nucleoside hydrolases that convert purine nucleotides and nucleosides, including hypoxanthine and guanine, into intermediate metabolites, effectively hindering the de novo synthesis of UA [5,6]. Second, specific LAB strains have been shown to influence host enzyme activity, particularly by inhibiting hepatic xanthine oxidase (XOD), which reduces endogenous UA production. Moreover, the short-chain fatty acids (SCFAs) generated during LAB fermentation play a vital role in preserving intestinal barrier function [7,8,9].

Therefore, LAB are increasingly being investigated for their uric acid-lowering properties in two complementary contexts: as potential probiotics that may confer direct health benefits when administered to the host, and as functional starter cultures that can enhance the technological properties of fermented foods. For instance, Lee et al. demonstrated that *Lacticaseibacillus paracasei* MJM60396 can degrade inosine and guanosine, reduce serum UA by 35%, and decrease hepatic XOD activity by 81%, alongside significant improvements in renal function and intestinal barrier integrity [7]. Similarly, *L. paracasei* 259 from yak yogurt suppressed hepatic XOD activity, thereby mitigating endogenous UA production and alleviating hyperuricemia symptoms [10]. Despite these promising findings, the current research focuses on the UA-lowering capabilities of probiotics under optimal or mesophilic conditions (37 °C). Such conditions may not accurately reflect the functional stability of these strains in real-world food processing environments, especially in cold regions. In practice, many fermented foods containing probiotics undergo low-temperature fermentation or cold storage, where microbial metabolic activity and enzyme kinetics are substantially altered. Indeed, low temperatures can both reduce the production of functional metabolites by probiotics and inhibit the growth of undesirable microorganisms, thereby affecting product safety and stability [11,12]. Consequently, there is a critical knowledge gap regarding the selection and evaluation of probiotic strains that maintain high UA-degrading activity at low temperatures, an essential consideration for the development of functional foods designed for cold-chain distribution.

The present study aims to address this critical research gap by systematically screening and isolating probiotics from traditional Northeast China kimchi, a fermented food matrix known for its microbial diversity and adaptability to low-temperature conditions. This study was designed with two complementary objectives. First, to identify strains capable of maintaining efficient and sustained uric acid degradation at temperatures representative of cold fermentation and refrigerated storage (10–15 °C), thereby serving as functional starter cultures for low-temperature fermented foods. Second, to evaluate the potential health benefits of the isolated strains, their uric acid-degrading capacity was also assessed at 37 °C, which simulates the human body temperature, supporting their potential as probiotics for hyperuricemia management. This dual-purpose approach enables the selection of strains suitable for low-temperature food fermentation that retain functionality under physiological conditions. Importantly, the application of the isolated strain in kimchi fermentation was designed to evaluate its technological functionality, including acid production, nitrite suppression, and sensory improvement, rather than to degrade uric acid in the food product, as uric acid is not a typical component of Kimchi. Thus, the isolated strain was employed to enhance the fermentation process and quality of Kimchi “*paocai*”, thereby providing a valuable resource for the development of low-temperature, UA-lowering functional foods.

## 2. Results

### 2.1. Identification of Cold-Adapted Probiotic Uric Acid-Degrading

**Figure 1:**
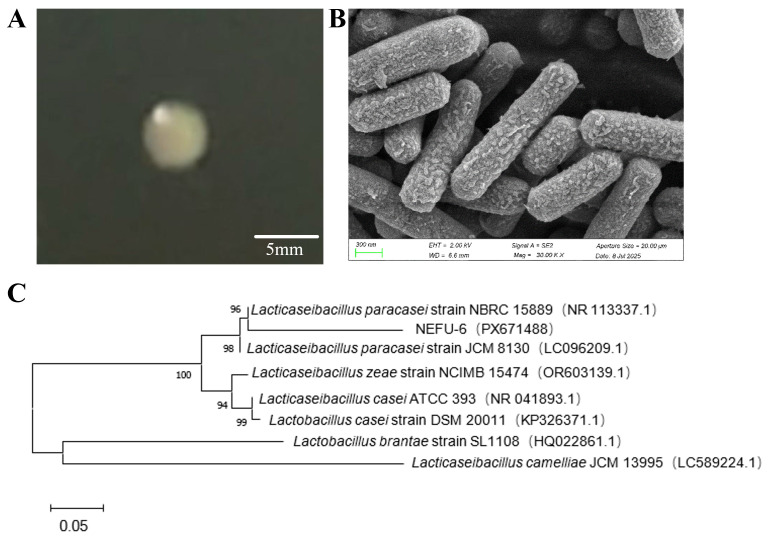
Identification of Strain NEFU-6 (**A**) Colony morphology, (**B**) Electron microscope image, and (**C**) Phylogenetic tree based on 16S rRNA gene sequence.

Using Northeast Chinese *paocai* as the sample source, an inorganic salt–uric acid medium was employed for bacterial enrichment. After 8 days of incubation at 15 °C, colonies with distinct morphologies were selected, resulting in the isolation of six individual strains. Among these, strain NEFU-6 exhibited significantly higher growth rates and did not produce spores. Biochemical characterization revealed that strain NEFU-6 was catalase-negative, unable to liquefy gelatin, and did not produce hydrogen sulfide, consistent with the metabolic profile of LAB. These traits, along with its Gram-positive and rod-shaped morphology, provisionally place it within the LAB group. Strain NEFU-6 formed small, milky-white colonies with entire margins and smooth surfaces on the MRS agar medium (Figure 1A). Scanning electron microscopy (SEM) revealed short, slender rod-shaped cells with rounded ends, approximately 0.7 μm in length and 0.2 μm in diameter, typical of lactobacilli (Figure 1B). Phylogenetic analysis based on 16S rRNA gene sequences confirmed that strain NEFU-6 clustered most closely with *Lacticaseibacillus paracasei* (Figure 1C).

### 2.2. Growth and Uric Acid Degradation Capacity of Strain NEFU-6

**Figure 2:**
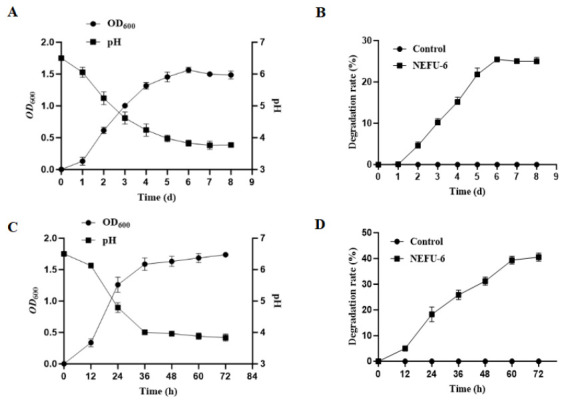
Growth, pH change, and uric acid degradation of strain NEFU-6 (**A**) pH profile and growth curve at 15 °C, (**B**) Uric acid degradation rate at 15 °C, (**C**) pH profile and growth curve at 37 °C, (**D**) Uric acid degradation rate at 37 °C.

Strain NEFU-6 displayed a typical growth curve at 15 °C. Following a 1-day lag phase, it entered exponential growth from day 1 to day 6, with OD600 reaching 1.56, before stabilizing in the stationary phase. Concurrent with growth, the strain acidified the medium, reducing the pH from 6.5 to 3.77 by day 8 (Figure 2A). The decline in pH was mainly synchronous with the increase in bacterial biomass. Uric acid degradation activity was first detected on day 1 (Figure 2B). The degradation rate increased to 25.48% on day 6. At 37 °C, the growth and metabolism of strain NEFU-6 were significantly accelerated (Figure 2C). The OD600 entered the exponential phase rapidly between 12 and 24 h and reached the stationary phase (OD600 = 1.59) by 36 h. Acidification occurred, with the pH decreasing from 6.5 to 4.79 within 24 h and to 3.83 by 72 h. Consistent with the faster growth, uric acid degradation between 60 and 72 h was 40.55 % (Figure 2D).

### 2.3. Safety Evaluation of Strain NEFU-6 (Indole, Hemolytic, and Gelatin Liquefaction Tests)

**Figure 3:**
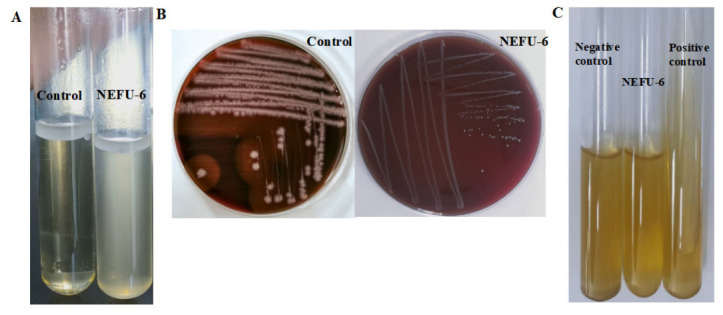
Safety parameters of strain NEFU-6 (**A**) Indole test results of strain NEFU-6 (**B**) Hemolysis and (**C**) Gelatin liquefaction test of strain NEFU-6.

Indole is a bacterial metabolite that can exert cytotoxic effects on host cells at elevated concentrations. Following the addition of Kovac’s reagent, no color change was observed in the medium’s surface layer compared with the blank control. The absence of red color development indicates a negative result: strain NEFU-6 does not produce tryptophanase during growth and is therefore incapable of catalyzing the breakdown of tryptophan to indole (Figure 3A). Hemolysins lyse red blood cells, contributing to anemia, tissue damage, and bacterial iron acquisition, key virulence mechanisms. The positive control strain exhibited clear β-hemolysis zones around its colonies.

In contrast, strain NEFU-6 showed normal growth without any clearing zones, confirming its lack of hemolytic activity (Figure 3B). The gelatin liquefaction activity of strain NEFU-6 was evaluated using gelatin medium. The positive control tube showed obvious liquefaction of the gelatin medium, whereas no significant changes were observed on the medium surface in the negative control and strain NEFU-6 tubes, which remained solid. These results indicate that strain NEFU-6 lacks gelatinase activity, further supporting its biosafety as a candidate probiotic strain (Figure 3C).

### 2.4. In Vitro Probiotic Properties of Strain NEFU-6

#### 2.4.1. Tolerance to Artificial Bile Salt

**Figure 4:**
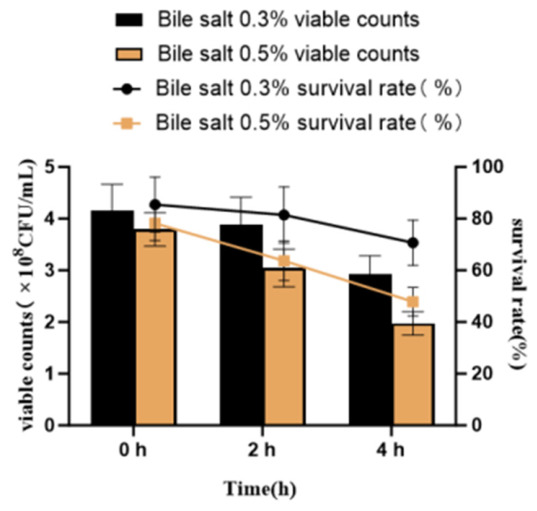
Bile salt tolerance of strain NEFU-6 at different bile salt concentrations. Viable counts (×10^8^ CFU/mL, bars) and survival rates (%, lines) of strain NEFU-6 after exposure to 0.3% (black) and 0.5% (orange) bile salts for 0, 2, and 4 h. Data are presented as means ± SD replicate.

The bile salt tolerance of strain NEFU-6 was evaluated at concentrations of 0.3% and 0.5%. At 0.3% bile salt, the average survival rate was 85.50% at 0 h, 81.45% at 2 h, and remained at a high level at 70.7% after 4 h of incubation. At the higher concentration of 0.5%, strain NEFU-6 maintained a relatively high survival rate of 63.74% within 2 h (Figure 4). These results demonstrate that NEFU-6 possesses excellent bile salt tolerance.

#### 2.4.2. Artificial Gastric Juice Analysis of Strain NEFU-6

**Figure 5:**
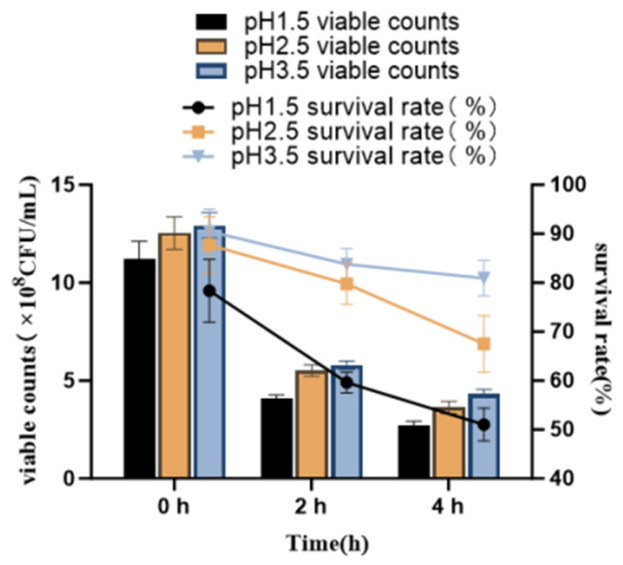
Survival rate of strain NEFU-6 in simulated gastric fluid.

The tolerance of strain NEFU-6 to simulated gastric fluid was evaluated over a range of pH values. After 4 h of exposure to pH 1.5, the strain NEFU-6 maintained a survival rate of 51.02%, indicating strong resistance to highly acidic conditions. At pH 2.5, the survival rate reached 67.54%, and at pH 3.5, it increased further to 80.94% after 4 h (Figure 5). These results indicate that strain NEFU-6 exhibits robust gastric acid tolerance, with survival progressively improving in more mildly acidic environments.

#### 2.4.3. Tolerance to Simulated Intestinal Fluid

**Figure 6:**
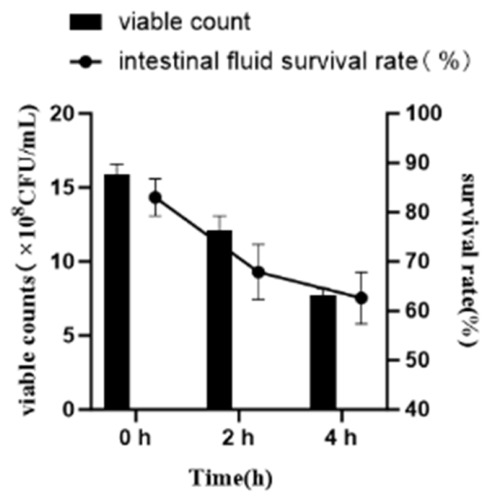
Survival rate of strain NEFU-6 in simulated intestinal fluid.

After 2 h of exposure, the survival rate of strain NEFU-6 reached 67.88%, and after 4 h, it remained at 62.61% (Figure 6). These results indicate that strain NEFU-6 exhibits promising intestinal tolerance.

#### 2.4.4. Antioxidant and Adhesion Properties of Strain NEFU-6

**Figure 7:**
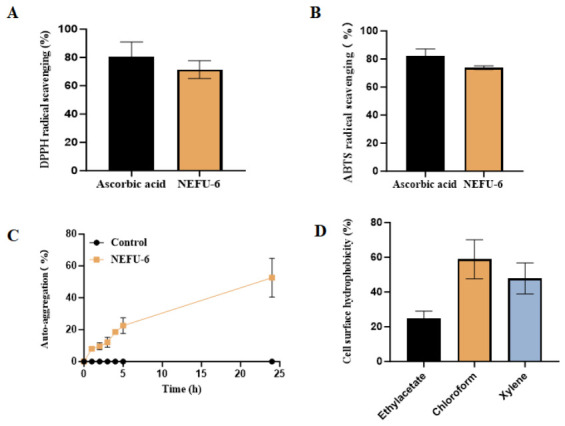
Evaluation of the in vitro probiotic characteristics of strain NEFU-6 (**A**) DPPH radical scavenging rate, (**B**) ABTS radical scavenging rate, (**C**) Assessment of strain auto-aggregation ability, and (**D**) Measurement of cell surface hydrophobicity.

The DPPH radical scavenging rate of strain NEFU-6 averaged 71.49%, comparable to that of the positive control ascorbic acid (80.46%) (Figure 7A), indicating strong antioxidant capacity. Similarly, the ABTS radical scavenging rate was 73.79%, approaching the 82.12% observed for vitamin C (Figure 7B). These results demonstrate that strain NEFU-6 possesses significant antioxidant potential, helping protect the intestinal environment from oxidative damage. Strain NEFU-6 auto-aggregation ability was increased significantly over time, rising from 8.06% at 1 h to 52.65% within 24 h (Figure 7C). This time-dependent increase suggests that strain NEFU-6 has a good capacity for auto-aggregation, which may facilitate the formation of biofilm-like structures and enhance its colonization and persistence in the intestinal tract.

The surface hydrophobicity of strain NEFU-6, assessed by microbial adhesion to solvents, was relatively high in chloroform (58.87%) and xylene (47.86%), indicating non-polar surface characteristics that favor adhesion to intestinal epithelial cells (Figure 7D). The value obtained using ethyl acetate was lower; however, the overall hydrophobicity profile suggests that strain NEFU-6 has potential for intestinal colonization.

### 2.5. Supplementation of Strain NEFU-6 Promotes Kimchi Fermentation

#### 2.5.1. Changes in Physicochemical Properties During Kimchi Fermentation

**Figure 8:**
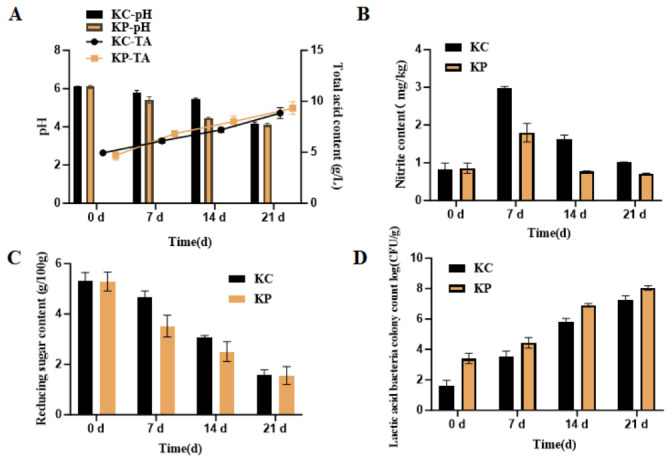
Changes during the fermentation of Kimchi by strain NEFU-6: (**A**) changes in pH value and total acid, (**B**) nitrite, (**C**) reducing sugar, and (**D**) lactic acid bacteria count. KP (Kimchi inoculated with strain NEFU-6), KC (naturally fermented Kimchi).

During fermentation, the pH of the kimchi samples decreased. The KP group (inoculated with *L. paracasei* NEFU-6) showed a pH of 5.40 ± 0.17, compared to 5.73 ± 0.13 in the KC group (natural fermentation without inoculation). Whereas, on day 14, there was a significant difference between the KP group (4.45 ± 0.08) and the KC group (5.41 ± 0.07) (*p* < 0.05). After 21 days of fermentation, both groups reached their lowest pH values, which were comparable and showed no significant difference. Correspondingly, the total acidity increased over time; the total acid content in the KP group (6.83 ± 0.32 g/kg) was higher than that of the KC group (6.08 ± 0.32 g/kg) on the 7th day. Furthermore, the KP group (8.02 ± 0.50 g/kg) exhibited a higher total acid content as compared to the KC group (7.09 ± 0.16 g/kg) (*p* < 0.05). At the end of fermentation (day 21), the KP group (9.33 ± 0.62 g/kg) still maintained a higher total acid content compared to the KC group (9.06 ± 0.39 g/kg) (Figure 8A). These findings indicate that inoculation with strain NEFU-6 significantly accelerated acid production during mid-fermentation, rapidly lowering the pH. This prompt acidification likely inhibited undesirable microbes and promoted earlier development of mature fermented flavors.

The nitrite content dynamics showed an initial increase, followed by a decline, in both the KC and KP groups. On day 7, the KC group reached its peak nitrite concentration (2.97 ± 0.04 mg/kg), which was significantly higher than that of the KP group (1.91 ± 0.20 mg/kg) (*p* < 0.05). Thereafter, nitrite levels decreased in both groups, yet the KC group consistently maintained higher nitrite concentrations than the KP group at days 14 and 21 (Figure 8B). These findings indicate that inoculation with strain NEFU-6 effectively suppressed nitrite accumulation during kimchi fermentation, particularly at the critical peak-formation stage on day 7.

Similarly, the reducing sugar content (3.52 ± 0.44 g/100 g) in the KP group on the 7th day was significantly lower than that of the KC group (4.65 ± 0.26 g/100 g). Moreover, no significant differences were observed between the groups at the end of fermentation (*p* > 0.05). This trend reflects the metabolic activity of LAB, which utilizes carbon sources to produce primarily lactic acid. The faster consumption of reducing sugars in the KP group during the early fermentation stage corresponds with the observed higher total acid content and more rapid pH decline (Figure 8C).

LAB counts were significantly higher in the KP group during the early fermentation stage (3.43 ± 0.48 log CFU/g) compared to the KC group (1.71 ± 0.47 log CFU/g) (*p* < 0.05). By day 7, LAB numbers (3.35 ± 0.44 log CFU/g) had increased from day 0 of fermentation (Figure 8D). The higher initial LAB in the KP group led to a significantly higher initial bacterial load, allowing them to become the dominant microbial population. This, in turn, helped suppress undesirable microbes and accelerated the development of mature fermented flavors.

#### 2.5.2. Quantification of Strain NEFU-6 in Kimchi Fermentation

**Figure 9:**
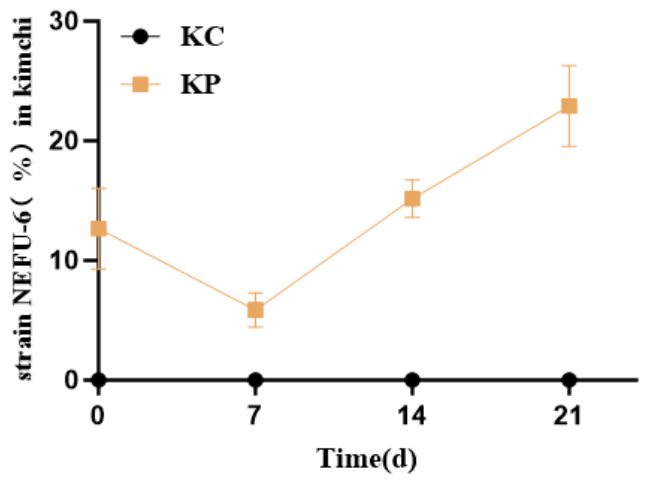
The absolute content (%) of strain NEFU-6 during the fermentation of Kimchi. KP (Kimchi inoculated with strain NEFU-6), KC (naturally fermented Kimchi).

The population dynamics of strain NEFU-6 during kimchi fermentation were monitored using absolute quantitative PCR (qPCR). The results showed that the relative abundance of NEFU-6 was 12.65% on day 0, suggesting an initial adaptation phase. This proportion declined significantly to 5.87% by day 7, indicating a transient reduction. However, by day 14, the abundance of NEFU-6 increased to 15.16%, and by the end of the fermentation process (day 21), it peaked at 22.92%, reflecting its increased relative abundance within the fermentation ecosystem (Figure 9). This data suggested that significantly higher counts in the inoculated group support the contribution of viable NEFU-6 cells to the fermentation process.

#### 2.5.3. Sensory Evaluation of Kimchi Fermented with Strain NEFU-6

**Figure 10:**
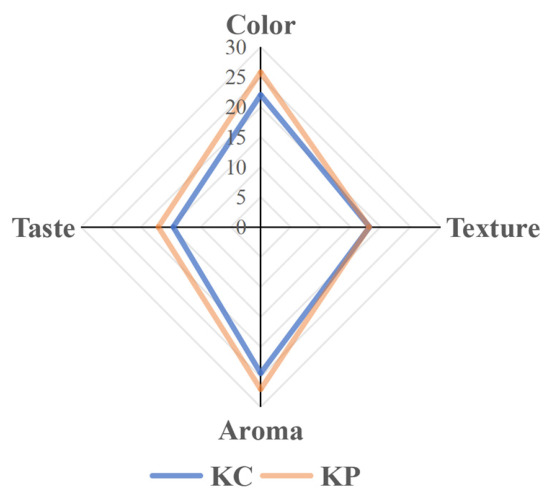
Sensory evaluation scores of Kimchi fermented with strain NEFU-6. KP (Kimchi inoculated with strain NEFU-6), KC (naturally fermented Kimchi).

Sensory evaluation was conducted to compare naturally fermented Kimchi (KC) and Kimchi inoculated with strain NEFU-6 (Group KP). In terms of color, Group KP achieved a significantly higher score (25.8 ± 1.3) than the KC group (22.1 ± 1.6) (*p* < 0.001), indicating that inoculation with NEFU-6 resulted in a brighter and more appealing appearance. For aroma, Group KP also outperformed KC (27.1 ± 1.4 vs. 24.5 ± 1.7, *p* < 0.001), indicating a richer, more intense fermented kimchi aroma. In the taste evaluation, Group KP scored significantly higher (16.8 ± 1.2) than KC (14.5 ± 1.5; *p* < 0.001), suggesting that the inoculated Kimchi had a clearer, fresher flavor profile, without bitterness or excessive saltiness. However, no significant difference in texture was observed between the two groups (Group KP: 18.1 ± 1.1; KC: 18.1 ± 1.2; *p* > 0.05), as both groups displayed comparable crispness, a characteristic inherent to Kimchi (Figure 10). These results demonstrate that the inoculation with strain NEFU-6 effectively enhances the sensory quality of Kimchi, particularly improving its visual appeal, aromatic intensity, and flavor freshness.

#### 2.5.4. Microbial Community Dynamics During Kimchi Fermentation with Strain NEFU-6

##### Bacterial Community Structural Shifts at Phylum and Genus Levels

**Figure 11:**
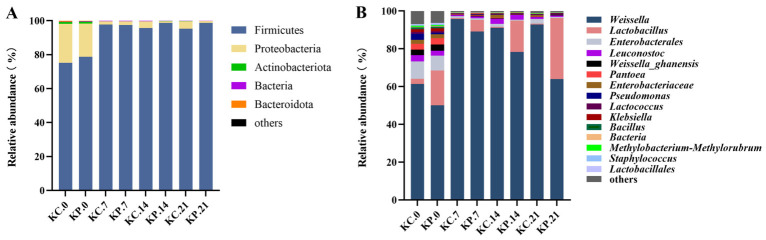
The bacterial community composition at the phylum (**A**) and genus (**B**) levels during kimchi fermentation.

At the phylum level (Figure 11A), the initial microbial structures of the experimental group (KP.0) and the control group (KC.0) were relatively similar, both dominated by Firmicutes (KC: 75.23%, KP: 78.83%) followed by Proteobacteria (KC: 22.90%, KP: 19.50%). Starting on day 7, a marked shift occurred in both groups, with the relative abundance of Firmicutes rapidly rising to dominate the ecosystem (KC: 97.74%, KP: 97.51%). This surge indicates the initiation of a typical lactic acid fermentation, which subsequently suppressed the growth of other phyla. Throughout the later fermentation stages (days 14–21), Firmicutes remained the dominant phylum (>95%) in both groups, albeit with slightly higher relative abundance in the experimental group.

At the genus level (Figure 11B), the relative abundance of *Lactobacillus* in the experimental group (KP) significantly surpassed that of the KC during the later fermentation stages (days 14–21). Notably, on day 21, the *Lactobacillus* abundance in the experimental group reached 32.28%, compared to only 2.45% in the KC, confirming successful colonization and dominance of the inoculated NEFU-6 strain, which aligns perfectly with qPCR findings. In the natural fermentation control, *Weissella* remained the absolute dominant genus throughout, consistent with traditional kimchi fermentation patterns reported in the literature. However, following NEFU-6 inoculation, the microbial community in the experimental group underwent a fundamental transformation, with both lactobacilli and *Weissella* emerging as dominant taxa. This shift demonstrates that probiotic inoculation can steer the fermentation process and shape a specific microbial community. Moreover, compared with the KC group, the experimental group exhibited consistently low levels of potential spoilage bacteria, including *Pseudomonas* and *Klebsiella*, across all time points. This suggests that NEFU-6 effectively suppressed the proliferation of these undesirable microbes through rapid acid production and competitive exclusion, thereby enhancing the safety and stability of the fermented product.

##### Alpha Diversity of the Microbial Community in Kimchi Fermented with Strain NEFU-6

The α-diversity indices of the kimchi samples were evaluated using QIIME 2, and the results are presented in Table 1. The Chao1 index, which estimates species richness, indicated that both the control and experimental groups possessed relatively high species richness during the early stages of fermentation. However, as fermentation progressed into the later stages, species richness in the experimental group (KP) was significantly lower than in the KC group, suggesting that the natural fermentation process (KC) retained a higher number of species towards the end of fermentation. In contrast, the Shannon index, which integrates both richness and evenness to reflect overall diversity, consistently remained higher in the KP across all time points, with a particularly pronounced difference observed on day 14. This finding implies that inoculation with NEFU-6 sustained a higher microbial diversity throughout the fermentation process. Additionally, the Pielou evenness index, which measures the uniformity of species distribution within a community, indicated that the KP exhibited greater evenness during the mid- to late-stage fermentation. This suggests that the microbial community in the NEFU-6 inoculated group was more evenly distributed.

##### Beta Diversity of the Microbial Community in Kimchi Fermented with Strain NEFU-6

**Figure 12:**
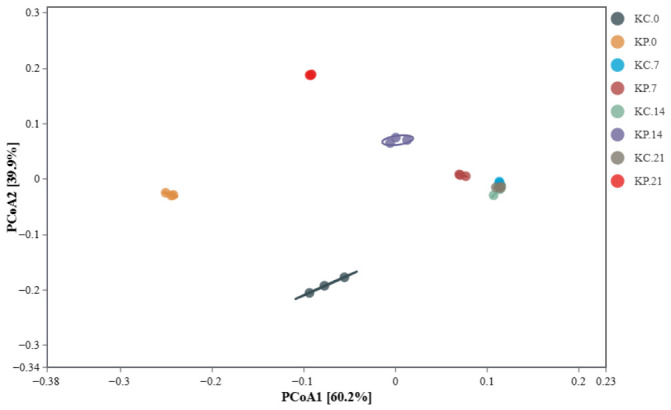
PCoA plot of different treatment groups during the fermentation of Kimchi. KP (Kimchi inoculated with strain NEFU-6), KC (naturally fermented Kimchi).

Beta diversity was assessed using Principal Coordinates Analysis (PCoA) based on distance matrices derived from the sequencing data. The first two principal coordinates (PCoA1 and PCoA2) accounted for the largest proportion of variance in microbial composition. Spatial proximity in the PCoA plot indicates similarity in community structure. The analysis revealed that samples from the KC group (days 7–21) clustered tightly together, indicating a stable, consistent microbial composition throughout natural fermentation. In contrast, samples from the KP group showed greater temporal dispersion, indicating significant shifts in microbial community structure following NEFU-6 inoculation (Figure 12).

## 3. Discussion

The rising global prevalence of hyperuricemia and gout, attributed to evolving dietary patterns, has emerged as a significant public health concern. In response, recent research has increasingly focused on LAB’s capacity to degrade purine compounds [13], offering an alternative approach for dietary interventions to reduce UA levels.

Therefore, screening for UA-lowering LAB has been conducted at 37 °C. In contrast, this study used low-temperature fermented kimchi from Northeast China as the sample source. It isolated the pure-cultured strain NEFU-6 at 15 °C, achieving a uric acid degradation rate of 25.48% under these conditions (Figure 2B). Notably, when evaluated at a simulated human intestinal temperature (37 °C), the strain NEFU-6 maintained a degradation rate of 40.55%, demonstrating broad temperature adaptability, a key attribute for potential in vivo functionality (Figure 2D). This dual-temperature evaluation strategy aligns with the two complementary objectives of the present study: (i) to select a functional starter culture suitable for low-temperature kimchi fermentation, and (ii) to assess the strain’s potential as a probiotic for hyperuricemia management.

The rationale for screening at 15 °C is based on the actual fermentation conditions of traditional Kimchi in Northeast China, where low-temperature fermentation (10–15 °C) is commonly practiced during winter months and refrigerated storage [14]. Importantly, in the field of food fermentation microbiology, “low temperature” refers to temperatures below the optimal growth range of mesophilic LAB (30–37 °C), rather than freezing temperatures [15]. Recent studies have systematically demonstrated that isolation temperatures of 10–20 °C are suitable for isolating psychrotrophic LAB from Kimchi, whereas 30 °C is unsuitable for isolating such strains [14]. Furthermore, elevated fermentation temperatures of 10–15 °C significantly enhance LAB diversity and metabolic activity compared to 4 °C, leading to more rapid acidification and distinct bacterial succession patterns [16]. Therefore, the 15 °C isolation temperature used in this study is appropriate for selecting cold-adapted strains that maintain functional activity under cold fermentation conditions and exhibit broad temperature adaptability for potential probiotic applications.

This finding is particularly significant, as it suggests that NEFU-6 may retain its functionality effectively within the human body. This was consistent with the previous report, which showed that *L. paracasei* Ma2 from healthy male feces achieved a UA degradation rate of 45.53% at 37 °C [17]. The difference in degradation performance between the two strains may be attributed to variations in sample origin and incubation temperature, underscoring the influence of environmental conditions on microbial uric acid-metabolizing capacity. It is important to note that this comparison was based on in vitro data from independent studies conducted under different experimental conditions. A direct in vivo comparison under controlled conditions was beyond the scope of the present study, as our primary objective was to isolate and characterize a cold-adapted uric acid-degrading LAB strain suitable for functional food fermentation, with a focus on its in vitro probiotic properties and application in kimchi fermentation. As outlined in the Future Research section, in vivo experiments using hyperuricemia animal models are planned to validate the functional efficacy of strain NEFU-6 and enable direct comparison with other reported strains.

In addition, the study monitored pH changes during fermentation. Strain NEFU-6 reduced the pH from an initial value of 6.5 to 3.77 by the end of fermentation, reflecting robust metabolic activity and proliferation. It was reported that pH declined from 6.7 to 4.2 using *L. paracasei* LcS under similar conditions [18]. Quantitative data further supported the more pronounced acidification observed with NEFU-6 during the mid-fermentation stage. Specifically, on day 7, the pH of the KP group (5.40 ± 0.17) was lower than that of the KC group (5.73 ± 0.13), and the total acid content of the KP group (6.83 ± 0.32 g/kg) was significantly higher than that of the KC group (6.08 ± 0.32 g/kg). On day 14, the difference became more pronounced, with the KP group reaching pH 4.45 ± 0.08 compared to pH 5.41 ± 0.07 in the KC group (*p* < 0.05), and total acid content in the KP group (8.02 ± 0.50 g/kg) was significantly higher than that of the KC group (7.09 ± 0.16 g/kg). These findings indicate that strain NEFU-6 exhibits faster growth and stronger acid-producing capacity, both desirable characteristics for fermentation starters. Such rapid acid production can effectively suppress spoilage and pathogenic microorganisms, thereby enhancing the stability and safety of fermented products. Importantly, the application of strain NEFU-6 in kimchi fermentation was designed to evaluate these technological functionalities, including acid production, nitrite suppression, and sensory improvement—rather than to degrade uric acid in the food product, as uric acid is not a typical component of Kimchi nor does it accumulate to significant levels during fermentation.

In the kimchi fermentation system, the persistence of strain NEFU-6 was monitored by qPCR targeting its specific DNA sequences (Figure 9). However, it is important to note that DNA-based qPCR cannot distinguish between viable and dead cells and therefore cannot directly demonstrate the strain’s metabolic activity in the food matrix. To complement this limitation, viable LAB counts (CFU/g) were also monitored throughout fermentation (Figure 8D), and the significantly higher counts in the inoculated group support the contribution of viable NEFU-6 cells to the fermentation process. Nevertheless, future studies should employ RNA-based analysis (e.g., RT-qPCR targeting mRNA) to directly assess the metabolic activity of the inoculated strain during kimchi fermentation, as recommended by previous studies.

Differences in gut microbiota composition between hyperuricemia patients and healthy individuals suggested the crucial role of the gut microbiota in regulating serum uric acid concentration and distribution [19,20,21]. Animal experiments further demonstrate that hyperuricemia can alter gut microbiota structure, while supplementation with specific probiotics can effectively reduce serum uric acid levels in hyperuricemia model mice [22,23]. However, the actual colonization of LAB in the gut involves complex factors such as adhesion and intestinal peristalsis, which may further promote uric acid breakdown [24]. In this context, auto-aggregation and surface hydrophobicity have been recognized as key in vitro indicators of bacterial adhesion and colonization potential. Stojanov et al. [25] demonstrated that vaginal lactobacilli with high surface hydrophobicity exhibited stronger adhesion to Caco-2 epithelial cells, and such adhesion correlated with their auto-aggregation properties. In addition, the safety of lactobacilli was also addressed in their study through hemolytic activity and Caco-2 cell viability assays, confirming the non-hemolytic nature and favorable safety profile of the tested strains. These findings support the use of these parameters to assess the colonization capability of probiotic strains preliminarily. This study systematically evaluated the strain’s in vitro functional characteristics, including comprehensive safety assessments, with its excellent auto-aggregation and strong surface hydrophobicity serving as preliminary evidence of its colonization potential; however, its colonization ability, survival rate, and uric acid-lowering efficacy in the actual intestinal environment require further validation through in vivo experiments. Antibiotic resistance profiling, recommended by FAO/WHO and EFSA guidelines for probiotic safety evaluation, was not performed in this study due to resource limitations and will be addressed in future studies. It is important to acknowledge that in vivo results may differ significantly from in vitro findings due to the complex physiological environment of the gastrointestinal tract, including host immunity, microbial interactions, and intestinal peristalsis, which cannot be fully replicated in vitro. Thus, strain NEFU-6 was employed in kimchi fermentation to enhance the health-beneficial properties of the functional food.

Future research should focus on the following key areas. First, animal models of hyperuricemia should be used to investigate the in vivo regulatory effects of oral administration of strain NEFU-6 fermented Kimchi. Second, the specific metabolic pathways and key enzymes involved in NEFU-6-mediated uric acid degradation will be further elucidated. These efforts will provide valuable strain resources and more direct scientific evidence for the development of low-temperature fermented foods or probiotic formulations enriched with uric acid-degrading functionality. The novelty of this approach lies not in low-temperature fermentation per se, but in the successful isolation and application of a *L. paracasei* strain that maintains both uric acid-degrading activity and probiotic functionality under low-temperature conditions (15 °C). By integrating the dual objectives of developing functional starter cultures and evaluating probiotic potential, this study provides a strain resource that bridges the gap between cold-chain food processing and the growing demand for functional foods with uric acid-lowering benefits. This strain fills a critical gap by enabling functional fermentation in cold environments, where conventional probiotic strains lose efficacy.

## 4. Materials and Methods

### 4.1. Media

Modified MRS medium (MRS supplemented with 0.84 g/L uric acid) [26] (Beef 10 g, diammonium hydrogen citrate 2 g, sucrose 20 g, Tween80 1 mL, sodium acetate 5 g, K_2_HPO_4_·3H_2_O 2 g, MgSO_4_·7H_2_O 0.58 g, MnSO_4_·4H_2_O 0.25 g, UA 0.84 g, distilled water 1000 mL) and UA medium [27]. Mineral Salt Medium (MSM) (Na_2_HPO_4_·12H_2_O 1.71 g, KH_2_PO_4_ 0.3 g, NaCl 0.05 g, MgSO_4_·7H_2_O 0.05 g, CaCl_2_·2H_2_O 0.001 g, UA 2 g, distilled water 1000 mL) was used for screening of uric acid strain. All chemicals and reagents used in this study were purchased from Shanghai Macklin Biochemical Co., Ltd. (Shanghai, China).

### 4.2. Screening of Lactic Acid-Degrading Uric Acid Strains at Low Temperatures

#### 4.2.1. Isolation and Screening of Low-Temperature Lactic Acid Bacteria

The strain was isolated from a homemade traditional kimchi, and the sample was collected from Yanji, Jilin Province, Northeast China. The kimchi sample was prepared by natural low-temperature fermentation without the addition of commercial starter cultures and exhibited a typical fermented aroma at the time of collection.

For isolation, 1 g of the kimchi sample was mixed with 100 mL of sterile distilled water in a 250 mL conical flask. The flask was incubated at 15 °C with shaking at 200 rpm for 1 h, followed by standing for 30 min. The supernatant was then inoculated onto inorganic salt-uric acid solid medium at 10% (*v*/*v*), and the plates were incubated at 15 °C for 3–8 days [28]. Single colonies with distinct morphology were selected and purified by repeated streaking. The purified strains were inoculated into modified MRS liquid medium and cultivated at 15 °C for 8 days. After three consecutive passages, when the strains’ growth rates stabilized, subculturing was performed every 8 days thereafter. The uric acid degradation ability, biomass, and dynamic pH changes in the stably passaged strains were determined. All incubations were performed under microaerophilic conditions [26] (solid medium in standard Petri dishes without anaerobic systems; liquid cultures in flasks sealed with breathable film and incubated statically).

#### 4.2.2. Determination of the Uric Acid Degradation Strain’s Ability

The activated pure strain was inoculated into modified MRS liquid medium and incubated statically at 15 °C for 8 days. After cultivation, the fermentation broth was collected, centrifuged, and the supernatant filtered through a 0.22 μm membrane filter. The uric acid content was then determined by high-performance liquid chromatography (HPLC) [27]. The high-performance liquid chromatography system (Thermo Fisher Scientific, Waltham, MA, USA) was equipped with a Waters XBridge C18 column (Waters Corporation, Milford, MA, USA). The chromatographic conditions were as follows: detection wavelength, λ = 254 nm; mobile phase, 0.2% phosphoric acid (pH 2.5–3.0) and methanol at a ratio of 98:2 (*v*/*v*); flow rate, 1.0 mL/min; column temperature, 25 °C. The uninoculated culture medium served as the blank control. The uric acid degradation rate was calculated using the following formula:

Degradation rate (%) = (*C*_0_ − *C*_1_)/*C*_0_ × 100%

where *C*_0_ is the uric acid concentration in the blank control, and *C*_1_ is the uric acid concentration in the fermentation broth.

### 4.3. 16S rRNA Gene Sequencing and Phylogenetic Analysis

Genomic DNA was extracted from bacterial strains using a Bacterial DNA Rapid Extraction Kit (Fangzhou Biosafety Technology Co., Ltd., Beijing, China) following the manufacturer’s instructions. The 16S rRNA gene was amplified by polymerase chain reaction (PCR) with the universal bacterial primers 27F and 1492R. PCR was performed in a 50.0 μL reaction mixture containing genomic DNA, 10× PCR buffer with Mg^2+^, Taq DNA polymerase, dNTPs, primers 27F and 1492R, and ddH_2_O. The amplified PCR products were purified and subjected to commercial sequencing by Sangon Biotech Co Ltd. (Shanghai, China). Bacterial morphology was analyzed using a scanning electron microscope (SEM, Sigma 300; Carl Zeiss AG, Oberkochen, Germany). The 16S rRNA gene sequences were compared with reference sequences in the GenBank database using the BLAST algorithm version 2.16.0 on the NCBI website to identify the most closely related taxa. A phylogenetic tree was constructed using MEGA (version 11) software.

### 4.4. Evaluation of In Vitro Survival Ability and Probiotic Characteristics of Lactic Acid Bacteria

#### 4.4.1. Safety Evaluation

##### Indole Test

The indole production test was performed following the method of Xu T et al. [29] with slight modifications. The activated strain was inoculated into peptone water medium and incubated under static microaerophilic conditions at 37 °C for 48 h. Subsequently, 1 mL of ethyl ether was added, and the mixture was vortexed. After standing for 5 min to allow the ether layer to separate on the culture surface, 1 mL of Kovac’s indole reagent was slowly added along the tube wall. The development of a red color at the interface of the two layers was interpreted as a positive result, indicating indole production, while the absence of color change was considered negative. An uninoculated medium blank was used as the negative control.

##### Hemolysis Test

Hemolytic activity was assessed as described by Motey et al. [30]. The activated strain was streaked onto Columbia blood agar plates (Columbia blood agar medium, Jiangmen Kailin Trading Co Ltd., Guangdong, China) and incubated at 37 °C for 48 h. *Escherichia coli* (ATCC 35218) was used as the positive control. After incubation, the plates were examined for clear zones (hemolysis) around colonies. The appearance of a clear, colorless zone surrounding the colony indicated β-hemolysis (complete hemolysis), a greenish zone indicated α-hemolysis (partial hemolysis), and the absence of any zone indicated γ-hemolysis (no hemolysis).

##### Gelatin Liquefaction Test

The gelatin liquefaction test was conducted to assess gelatinase activity according to the method [31]. The test strain was inoculated into tubes containing gelatin medium, while sterile physiological saline served as the negative control. Tubes inoculated with *Bacillus subtilis* ATCC 6633 were used as the positive control. All tubes were incubated under appropriate conditions, and gelatin liquefaction was observed periodically. The positive control showed complete liquefaction, confirming the assay’s validity. Solidification of the medium upon refrigeration indicated the absence of gelatinase activity (negative result), whereas a liquid medium remaining after refrigeration indicated gelatinase activity and a positive result for gelatin liquefaction.

#### 4.4.2. Evaluation of Probiotic Properties

##### DPPH Radical Scavenging Assay

The DPPH radical scavenging activity of the strain was determined [32] with slight modifications. Briefly, 100 μL of bacterial suspension and 100 μL of 0.4 mmol/L DPPH solution (prepared in anhydrous ethanol) were added to a 96-well plate. A mixture of 100 μL of vitamin C (VC) solution and 100 μL of 0.4 mmol/L DPPH solution served as the positive control. The reaction mixture was incubated in the dark at 20 °C for 30 min. The absorbance was measured at 540 nm using a microplate reader. All experiments were performed in triplicate. The DPPH radical scavenging rate was calculated using the following formula:

DPPH% = [1 − (*B* ÷ *A*)] × 100

where *A* is the absorbance of the control, and *B* is the absorbance of the sample.

##### ABTS Radical Scavenging Assay

The ABTS radical scavenging activity was assessed following the method of Khan et al. [33]. Briefly, ABTS radical cations (ABTS^+^) were generated by reacting 7.4 mmol/L ABTS stock solution with 2.6 mmol/L potassium persulfate in the dark at room temperature for 24 h. Before analysis, the ABTS^+^ working solution was diluted with phosphate-buffered saline (PBS, pH 7.4) to an absorbance of 0.70 ± 0.03 at 734 nm. Subsequently, 20 μL of bacterial suspension was mixed with 180 μL of the diluted ABTS^+^ solution in a 96-well plate. Vitamin C (VC) was used as the positive control. The mixture was incubated in the dark at 20 °C for 10 min, and the absorbance was measured at 734 nm. All assays were conducted in triplicate. The ABTS radical scavenging rate was calculated as follows:

ABTS % = 1 − *B* ÷ *A* × 100

where *A* is the absorbance of the control, and *B* is the absorbance of the sample.

##### Auto-Aggregation Ability

The auto-aggregation ability of strain NEFU-6 was determined accordingly, with slight modifications [33]. The strain was cultured in MRS broth at 37 °C for 24 h. Cells were harvested by centrifugation, washed twice with sterile PBS, and resuspended in the same buffer. The cell suspension was vortexed for 30 s to ensure homogeneity. The absorbance at 600 nm (*A*_0_) was measured immediately. The suspension was then allowed to stand at room temperature, and the absorbance at 600 nm (*A*_t_) was measured at different time intervals (1, 2, 3, 4, 5, and 24 h). All measurements were performed in triplicate. The auto-aggregation percentage was calculated using the following formula:

Auto-aggregation % = (1 − *A*_t_/*A*_0_) × 100

where *A*_0_ represents the initial absorbance at 0 h, and *A* represents the absorbance at time t (1, 2, 3, 4, 5, or 24 h).

##### Cell Surface Hydrophobicity

The cell surface hydrophobicity of strain NEFU-6 was evaluated following the method of Reuben et al. [34] with slight modifications. The strain was cultured in MRS broth at 37 °C for 24 h. Cells were harvested by centrifugation, washed twice with sterile PBS, and resuspended in 5 mL of PBS. The initial absorbance at 600 nm was measured and recorded as *A*_0_. Equal volumes of the cell suspension and three different solvents, ethyl acetate (basic solvent), chloroform (acidic solvent), and xylene (non-polar solvent) were mixed by vortexing for 5 min. The mixture was then allowed to stand at room temperature for 30 min to achieve phase separation. The aqueous phase was carefully removed, and its absorbance at 600 nm was measured (*A*_t_). All experiments were performed in triplicate. The cell surface hydrophobicity was calculated using the following formula:

Hydrophobicity% = (1 − *A*_t_/*A*_0_) × 100

where *A*_t_ represented the absorbance of the aqueous phase after the two-phase separation, whereas that of the *A*_0_ represented the absorbance before mixing with the solvent.

#### 4.4.3. Bile Salt Tolerance Assay

Strain NEFU-6 was revived by two successive subcultures to ensure its viability and subsequently inoculated at 2% (*v*/*v*) into sterile MRS broth (control group) as well as into modified MRS broth containing 0.3% or 0.5% (*w*/*v*) bovine bile salt (Shanghai Macklin Biochemical Technology Co., Ltd., Shanghai, China) (treatment groups), respectively, and all inoculated media were statically incubated at 37 °C, with samples collected at 0, 2, and 4 h of incubation. All inoculated media were statically incubated at 37 °C, and samples were collected at 0, 2, and 4 h. Each sample was serially diluted with sterile physiological saline, and appropriate dilutions were spread onto MRS agar plates in triplicate. After incubation at 37 °C for 48 h, the number of viable colonies was counted, and the colony-forming units per milliliter were calculated. The growth status of LAB in the bile salt-containing media was compared with that in the control MRS broth, and the survival rate was computed using the following formula [35].

Survival rate (%) = (Viable count in bile salt-supplemented MRS broth/Viable count  in control MRS broth) × 100%.

#### 4.4.4. Simulated Gastric Juice Tolerance Assay

Simulated gastric juice was prepared according to the method described [36]. The pH of the simulated gastric juice was adjusted to 1.5, 2.5, and 3.5 to simulate strong gastric acidity in the fasting state, typical gastric conditions, and the diluted state after a meal, respectively. Two successive subcultures were performed, then the culture was inoculated at 2% (*v*/*v*) into MRS broth and cultivated at 37 °C with shaking at 180 r/min. Under sterile conditions, 5 mL of the bacterial culture was collected and centrifuged to retain the cell pellet. The pellet was washed with 4 mL of sterile physiological saline and centrifuged at 6000 r/min for 5 min at 4 °C, after which the supernatant was discarded. The pellet was then rinsed with 4 mL of 0.01 mol/L PBS (pH 7.4), centrifuged again, and the supernatant was removed. Finally, the pellet was resuspended in 5 mL of simulated gastric juice and 1.5 mL of PBS, and the pH was precisely adjusted to 1.5, 2.5, or 3.5 as required. The suspension was vortexed thoroughly and placed in a constant-temperature shaker at 37 °C. Samples were taken immediately (0 h) and after 2 h of incubation, appropriately diluted, and spread onto MRS agar plates for viable cell counting. A control group was prepared by diluting the same bacterial culture in sterile MRS broth without gastric juice and incubating it under the same conditions; viable counts were determined at the corresponding time points. All experiments were performed in triplicate, and the mean values were calculated. The survival rate was calculated using the following formula:

Survival rate (%) = (viable count in simulated gastric juice-treated group (CFU/mL)/viable  count in control MRS broth (CFU/mL)) × 100%.

#### 4.4.5. Simulated Intestinal Fluid Tolerance Assay

The strain’s tolerance to simulated intestinal fluid was evaluated according to the method described in the literature [37]. Simulated intestinal fluid was prepared according to the Chinese Pharmacopoeia (2020 edition, volume IV, general chapter 0921), containing 0.68 g/100 mL monopotassium phosphate (KH_2_PO_4_) and 1 g/100 mL trypsin (Shanghai Macklin Biochemical Technology Co., Ltd., Shanghai, China), with the pH adjusted to 6.8 using 0.1 mol/L NaOH. The activated strain NEFU-6 was cultured in liquid medium at 37 °C with shaking at 180 r/min using a 2% (*v*/*v*) inoculum. Under sterile conditions, 5 mL of the bacterial culture was collected and centrifuged to retain the cell pellet. The pellet was washed with 4 mL of sterile physiological saline and centrifuged at 6000 r/min for 5 min at 4 °C, and the supernatant was discarded. Subsequently, the pellet was rinsed with 4 mL of 0.01 mol/L PBS (pH 7.4), centrifuged again, and the supernatant was discarded. Finally, the pellet was resuspended in 6.5 mL of simulated intestinal fluid, and the pH was accurately adjusted to 6.8. The suspension was vortexed thoroughly and placed in a constant-temperature shaker at 37 °C. Samples were taken at 0, 2, and 4 h of incubation, appropriately diluted, and spread onto MRS agar plates for viable cell counting. The strain’s survival rate after 2 h of exposure was determined. All experiments were performed in triplicate, and the mean values were calculated. The survival rate was calculated using the following formula:

Survival rate (%) = (CFU/mL in simulated intestinal fluid-treated group)/(CFU/mL in  control group) × 100%.

### 4.5. Preparation of Fermented Kimchi

#### 4.5.1. Production Process of Fermented Kimchi and Enumeration of Lactic Acid Bacteria

The fermentation experiment was conducted in a controlled laboratory setting with two treatment groups, each consisting of three replicates: the KP group (inoculated with *L. paracasei* NEFU-6) and the KC group (natural fermentation without inoculation). Fresh Chinese cabbage (2.5 kg) was uniformly salted with 300 g of edible salt and dehydrated at room temperature for 12 h. The seasoning mixture, comprising 200 g each of apple and pear, 100 g each of white radish and onion, and 50 g each of ginger and garlic, was blended with a predetermined amount of chili powder, fish sauce, and shrimp paste into a homogeneous paste, which was then evenly applied to the dehydrated cabbage. The prepared cabbage was placed into 4 L glass fermentation vessels. *L. paracasei* NEFU-6 was activated through two successive subcultures, enriched, and concentrated via centrifugation to obtain a bacterial suspension with a concentration of approximately 10^8^ CFU/mL. For the KP group, the bacterial suspension was added at 3% of the total fermentation volume, resulting in an initial inoculation density of approximately 10^6^ CFU/mL; no bacterial suspension was added to the KC group. Both groups were sealed and fermented at 15 °C for 21 days. Samples were collected on days 0, 7, 14, and 21 for physicochemical analysis and microbial community profiling, and a final sensory evaluation was performed on day 21.

LABs were enumerated according to the method described in the literature [38] with slight modifications. At each sampling time point, 10 g of the kimchi sample (including both solid and liquid components) was homogenized with 90 mL of sterile physiological saline using a stomacher. The homogenate was serially diluted, and 100 μL of each dilution was spread onto MRS agar plates in triplicate. Plates were incubated at 37 °C for 48 h under microaerophilic conditions. Colonies showing typical morphological characteristics of LAB were counted, and results were expressed as colony-forming units per gram of kimchi sample (CFU/g).

#### 4.5.2. Physicochemical Analyses

The physicochemical properties of the fermented Kimchi were evaluated using the following standard methods. The pH was determined using a pH meter, following the method described by Karim et al. [39] (employing AOAC official method 981.12), and the total acid content was assessed via acid-base titration as described by Jeong et al. [40] Nitrite content was measured using the improved spectrophotometric method validated by Lim et al. [41], based on the diazo-coupling reaction, and the reducing sugar content was determined by the 3,5-dinitrosalicylic acid (DNS) colorimetric method, as reported [42]. LAB counts were enumerated by plate count on MRS agar, following the protocol established by Karaman et al. [43].

#### 4.5.3. Quantitative qPCR Analysis of Inoculated Strain NEFU-6 in Kimchi

To accurately assess the colonization ability of the inoculated strain in the final kimchi product, absolute quantitative real-time PCR (qPCR) was performed [44]. The primers used included strain-specific primers targeting *Lacticaseibacillus paracasei* NEFU-6 and universal bacterial primers targeting the 16S rRNA gene. The primer sequences were as follows: for specific detection of NEFU-6, F: 5′-GCATTAAGCATTCCGCCTGG-3′ and R: 5′-CCTGGTAAGGTTCTTCGCGT-3′; for universal bacterial quantification, F: 5′-AGAGTTTGATCCTGGCTCAG-3′ and R: 5′-TACGGCTACCTTGTTACGACTT-3′. First, strain-specific primers targeting unique gene sequences of the strain were designed and synthesized to ensure high specificity. An absolute quantification standard curve was then constructed: the target gene fragment of the strain was cloned into a plasmid vector, extracted, purified, and accurately quantified. A series of plasmid DNA dilutions with known copy numbers was prepared as standards.

After fermentation, total genomic DNA was extracted from the kimchi samples. The standards and sample DNA were amplified in the same reaction system using real-time fluorescence PCR (LightCycler 480 System, Roche Diagnostics, Basel, Switzerland) with SYBR Green Master Mix (Thermo Fisher Scientific, Waltham, MA, USA). By analyzing the Ct values of the samples against the standard curve, the absolute gene copy number of the strain per gram of Kimchi was calculated, directly determining its final content in the fermentation system.

#### 4.5.4. Sensory Evaluation

Sensory evaluation was conducted using a modified version of the sensory assessment model proposed by Kraouia M et al. [45]. A panel of 15 trained professionals, free of sensory disorders and common colds, performed blind scoring of the kimchi samples after 21 days of fermentation. The evaluation employed a segmented scoring system to assess four aspects: color and appearance, aroma, flavor, and texture, according to the criteria detailed in Supplementary Table S1. Data analysis was performed using SPSS 27.0, with statistical significance set at *p* < 0.05, and visualizations were generated using GraphPad Prism 9.0 (version 9.5.1) and SIMCA 14.1.

#### 4.5.5. Microbial Community Sequencing

After sampling, the kimchi fermentation samples were stored at −80 °C and subsequently outsourced to a professional sequencing service (Meige Biotechnology Co., Ltd., Wuhan, China) for high-throughput bacterial community analysis using the Illumina MiSeq platform. Raw sequencing data were processed through assembly, quality filtering, and optimization to obtain high-quality reads. Operational Taxonomic Units (OTUs) were clustered at a 97% similarity threshold using the Usearch version 11. Taxonomic classification was performed using Usearch version 11 (Uclust algorithm) with a confidence threshold of 0.8. Representative sequences for each OTU were then aligned against the NCBI (version 2.17.0) and SILVA (release 138) databases to obtain species-level annotations.

## 5. Conclusions

Strain NEFU-6 was identified as a cold-adapted uric acid-degrading strain. This strain was evaluated with two complementary objectives in mind. First, it exhibited a uric acid degradation rate of 25.48% after 6 days of cultivation at 15 °C, demonstrating its potential as a functional starter culture for low-temperature fermented foods. Second, NEFU-6 exhibited excellent probiotic properties in vitro, including strong bile salt tolerance, substantial gastric acid resistance, significant antioxidant activity, and cell-surface adhesion capacity, meeting the safety requirements for food applications and supporting its potential as a candidate probiotic for hyperuricemia management. In addition, when applied to kimchi fermentation, NEFU-6 effectively regulates microbial community structure, promoting *Lactobacillus* spp. as the dominant flora, accelerating acid production, inhibiting nitrite accumulation, and significantly enhancing the sensory quality of the Kimchi. These findings provide reliable strain resources and a theoretical basis for developing functional fermented foods.

## Figures and Tables

**Table 1:** Alpha Diversity Index of Different Treatment Groups During Kimchi Fermentation.

Group	Chao1	Pielou	Shannon
KC.0	278.00 ± 11.14	0.50 ± 0.01	4.09 ± 0.13
KP.0	253.00 ± 15.13	0.52 ± 0.00	4.11 ± 0.06
KC.7	97.00 ± 3.61	0.28 ± 0.00	1.83 ± 0.02
KP.7	95.33 ± 8.39	0.31 ± 0.01	2.04 ± 0.03
KC.14	106.00 ± 14.18	0.30 ± 0.02	1.99 ± 0.17
KP.14	80.67 ± 6.66	0.34 ± 0.03	2.17 ± 0.19
KC.21	116.67 ± 10.97	0.30 ± 0.01	2.08 ± 0.05
KP.21	81.33 ± 9.07	0.35 ± 0.01	2.19 ± 0.07

## Data Availability

The data presented in this study are available in this article and are available from the corresponding author upon reasonable request.

## Supplementary Materials

The following supporting information can be downloaded at: https://www.mdpi.com/article/10.3390/molecules31101717/s1, Table S1: Sensory evaluation table for Kimchi.
