# Supplementary material for: Cold-Adapted Uric Acid-Degrading Lacticaseibacillus paracasei NEFU-6 Application in Kimchi “Paocai”
Source: Molecules. 2026 May 18;31(10):1717. doi: 10.3390/molecules31101717 (PMC13210145; doi:10.3390/molecules31101717)
Supplement: Supplementary file 1 [file molecules-31-01717-s001.zip › molecules-4220644-supplementary.pdf]

Cold-Adapted Uric Acid-Degrading *Lacticaseibacillus paracasei* NEFU-6 Application in Kimchi "*Paocai*"

Xiaoyu Wang [1], Binyu Cui [2], Xiaoqian Zhou [2], Wei Zhang [2], Aman Khan [2], and Weidong Wang [1,2,*]

[1] Key Laboratory of Green and Low-Carbon Agriculture for Northeastern Plains, Ministry of Agriculture and Rural Affairs, College of Life Science and Technology, Heilongjiang Bayi Agricultural University,
Daqing 163319, China; wangxiaoyu12025@126.com

[2] College of Life Sciences, Northeast Forestry University, Harbin 150400, China; joycecc1231@163.com (B.C.); 17861233010@163.com (X.Z.); m19852575529@163.com (W.Z.); aman@lzu.edu.cn (A.K.)

* Correspondence: wdwang@nefu.edu.cn

Table S1. Sensory evaluation table for Kimchi

Evaluation Criteria	Sensory Description	Score / Points
Color (30 points)	Bright color with luster, uniform size, and no impurities.	20-30
	Relatively bright color with slight luster, relatively uniform size, and no impurities.	10-19
	Dull color without luster, uneven size, and a few impurities.	0-9
Aroma (30 points)	Very strong and rich fermented pickles aroma, no off-odors.	20-30
	Moderate fermented kimchiaroma, no off-odors.	10-19
	Weak fermented pickles aroma, no off-odors.	0-9
Taste (20 points)	Pure fermented pickles taste delicious and palatable, with no bitter, astringent, or overly salty aftertaste.	14-20
	Acceptable fermented pickles taste, no bitter, astringent, or overly salty aftertaste.	7-13
	Poor fermented pickles taste, with off-flavors such as bitterness, astringency, or excessive saltiness.	0-6
Texture (20 points)	Retains the inherent crispness of pickles, with a very crisp and tender texture, no softness or rot.	14-20
	Moderate crispness and tenderness, slight softness, no rot.	7-13
	No sense of crispness, soft, loose, crumbly, or even rotten.	0-6
